# The Characterization of Natural Resins and a Study of Their Degradation in Interactions with Zinc Oxide Pigment

**DOI:** 10.3390/ma17225416

**Published:** 2024-11-06

**Authors:** Elisa Calà, Alessandro Croce, Laura Cagna, Andrea Marangon, Giorgio Gatti, Maurizio Aceto

**Affiliations:** 1 Dipartimento per lo Sviluppo Sostenibile e la Transizione Ecologica, Università degli Studi del Piemonte Orientale, Piazza S. Eusebio 5, 13100 Vercelli, Italy; elisa.cala@uniupo.it (E.C.); andrea.marangon@uniupo.it (A.M.); giorgio.gatti@uniupo.it (G.G.); maurizio.aceto@uniupo.it (M.A.); 2 SSD Research Laboratories, Research and Innovation Department (DAIRI), Azienda Ospedaliero-Universitaria SS. Antonio e Biagio e Cesare Arrigo, Via Venezia 16, 15121 Alessandria, Italy; 3 Dipartimento di Scienze e Innovazione Tecnologica, Università degli Studi del Piemonte Orientale, Viale Teresa Michel 11, 15121 Alessandria, Italy; laura.cagna@uniupo.it

**Keywords:** IR spectroscopy, natural resins, colophony, mastic, thermal degradation, UV degradation

## Abstract

In the last few years, the role of science in Cultural Heritage has assumed greater significance since diagnostics have become essential for the characterization of artworks. The development of conservation strategies involves growing the study of artworks and the knowledge of the materials used against the degradation plaguing the painted surfaces. This work focuses on the investigation of the degradation processes involving paintings on canvas, in particular delamination and progressive deterioration of the painted surfaces. The main causes of the degradation are attributable to the formation of metal soaps, which originate from the interaction between binders and pigments; as a result, the process leads to the progressive fracturing of the paint film. Using various characterization techniques allowed us to acquire information on the structural and morphological properties of the binder resins and study the binder/pigment interaction during the degradation process to understand the quantity and quality of the acid sites present in the binders and, consequently, the potential reactivity with the cationic part of the pigments. The binders were also analyzed within paint layers in contact with zinc oxide to study the interactions and the possible formation of new species as metal soaps and metal oxalates that can modify the boundary among the painting layers and, consequently, the appearance of the artwork and its artistic value. Modifications after UV and thermal aging processes were observed using Infrared spectroscopy and thermogravimetric analysis. Zinc soap formation was observed after 7 h of a UV aging process and was correlated to the acidity of the resins.

## 1. Introduction

Natural resins are commonly and widely employed in the Cultural Heritage field and in conservation as finishing layers or varnishes due to their optical and preservative properties [1]. In many cases, they have been mixed with drying oils in order to obtain oleo-resinous binders with particular aesthetical properties, or else used purely as binders in retouching paintings [2]. In all these applications, the resins come in contact with pigments by direct mixing or by interaction with the underneath painting layer, which is often impoverished by natural binder migration.

The stability and degradation behavior of natural resins have been investigated by many authors for their important role in conservation chemistry, but compositions and related degradation issues are not completely understood [3,4,5,6,7].

The nature of resins is complex and, generally speaking, they are mixtures of isoprenic structures (terpenes) in which a polymeric fraction could be present [8,9,10]; in particular, they contain mono- and poly-esters and free acids. More specifically, Copal, Sandarac and Colophony are diterpenoid resins, while Dammar, Mastic and Shellac are mixtures of triterpenoid and different cyclic sesquiterpenoid acids [9,11].

It is well known that the presence of pigment dramatically influences the response of polymers and resins towards thermal and photo-oxidative stresses [5,6,12]. In this work, the study of degradation mechanisms of paint layers will be presented and, in particular, the aging behavior of commercial Colophony (Kremer Pigmente no. 60310) in the presence of white zinc (ZnO) will be described. It is now known how the presence of ZnO and resins leads to the formation of salts, over time, called zinc soaps [13,14,15,16,17]. The formation of zinc soaps brings to light the need for restoration actions. Many studies over the decades have focused on studying the formation of zinc soaps, but only a small number of these have investigated the effects on the resins used [12,18].

In addition, we studied the effect of thermal and solar aging simulation to try to evaluate the decay behaviors related to the different aging factors (temperature and UV-VIS irradiation), in order to increase knowledge to improve restoration and basic knowledge in the field of Cultural Heritage.

In the scientific literature [4,6,7,11,19], mixtures of a large number of paintings, binders and varnishes (drying oil and natural resins) with the most common historical pigment have been studied in different aging conditions, such as solar simulation, thermal simulation at 60 °C and thermal simulation at 300 °C. These studies had the goal of evaluating the influence of inorganic pigments in the degradation processes of organic materials, such as the formation of metal carboxylates. Subsequently, in this study, the characterization of the binders was performed by analysis using FTIR spectroscopy coupled with thermogravimetric analysis, with the aim of better understanding the chemical structures of the materials and their behavior in conditions of thermal degradation simulation. It was possible to detect the presence of special additives and then formulate a classification of the resins in categories based on the functional groups and distributions of the encountered weight losses. There was a renewed focus on the study of a single binder, the commercial Colophony 60310, which showed the presence of additives in its formulation, that may alter the reactivity that should be accelerating the degradation process. To better understand the effects, samples with pure Colophony added have been compared. Finally, we analyzed paint layers of Colophony or Mastic and ZnO in order to verify the photo-oxidative reactivity and the thermal degradation in real conditions.

Several conducted studies were focused on the zinc role in photo-degradation and in the formation of zinc oxalate or zinc salts after UV aging on various resins and polymer substrates [20,21,22,23,24,25]. The characterization of the resins before and after irradiation with sunlight and the formation of zinc salts is still very little studied [13,14,15,16,17]. This work highlights how the presence of ZnO and talc (an additive found in commercial formulations) can increase the rate of degradation of resins used in Cultural Heritage restoration, leading to the rapid formation of zinc soaps under UV irradiation.

## 2. Materials and Methods

All the natural resins (Manila Copal, Sandarac, Colophony, Dammar, Shellac and Mastic) and pure zinc oxide pigment were supplied by Kremer Pigmente GmbH & Co. (Aichstetten, Germany).

In order to simulate real degradation, acetone (Aldrich, St. Louis, MO, USA) solutions of the mixture Mastic/ZnO (1:1 in weight) and Colophony/ZnO (1:1) were applied by brush on polished silicon wafers that were used as support.

Two samples of mixtures were aged in the following ways:

- A total of 7 h of simulated solar irradiation with a UV solar lamp with an average irradiation of 1000 W/m^2^.
- In a FTIR apparatus where it was possible to vary the temperature from 30 °C to 500 °C of thermal aging.

### 2.1. FTIR Spectroscopy Analysis

FTIR spectra of materials were collected using a Thermo Electron Corporation (Thermo Fisher Scientific, Waltham, MA, USA) FT Nicolet 5700 spectroscope; the resolution of the obtained spectra was 4 cm^−1^. The samples were analyzed under vacuum conditions, using a quartz IR cell equipped with KBr windows attached to a vacuum line, with a residual pressure of ≤1 × 10^−4^ mbar at different temperatures.

### 2.2. Thermogravimetric Analysis

Analysis was performed on a Setaram (Philadelphia, PA, USA) SETSYS Evolution instrument under argon (gas flow 20 mL/min), heating the resin samples up to 500 °C at a rate of 5 °C/min.

### 2.3. Scanning Electron Microscopy (SEM)

SEM images were recorded on a Quanta 200 FEI (Hillsboro, OR, USA) Philips scanning electron microscope equipped with an EDAX EDS attachment, using a tungsten filament as an electron source at 20 keV.

## 3. Results

### 3.1. Thermogravimetric Analysis of Resins

**Figure 1:**
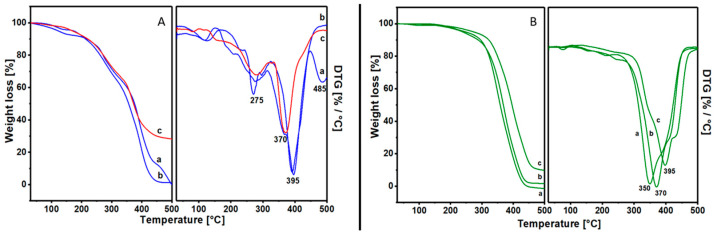
(**A**) left, comparison of the thermogravimetric analysis of the samples: (a) Manila Copal 60150, (b) Sandarac 60100, (c) Colophony 60310; right, the related derivative (DTG). (**B**) left, comparison of the thermogravimetric analysis of the samples: (a) Dammar 60000, (b) Mastic 60050, (c) Shellac 60410; right, the related derivative (DTG).

Figure 1A,B report the thermogravimetric analysis of all samples. In particular, the analyses of Manila Copal, Sandarac and Colophony samples show similar thermal behavior (Figure 1A), as well as the analyses of Dammar, Mastic and Shellac samples (Figure 1B). In addition, the loss in weight is very similar in both the class of materials.

In the first class (Figure 1A), all three resins have a similar distribution of loss in weight, with an initial non-uniform distribution of a slight decline weight and a significant weight loss from 350 °C to 500 °C. In this case, only Colophony (c in Figure 1A) shows a percentage of residual material at 500 °C, which is due to the presence of additives in the resin.

The thermogravimetric analysis of the second category, consisting of Dammar (a), Mastic (b) and Shellac (c), is represented in Figure 1B.

All these materials have a first weight loss at around 100 °C (about 1 wt%) associated with dehydration processes related to traces of water and a further loss centered at 350, 370 and 395 °C for Dammar, Mastic and Shellac, respectively. Even in this case, the Shellac sample has a weight loss that is not complete at 500 °C; this is due to a different chemical composition and, in particular, it should be attributed to the presence of sesquiterpene species [26,27,28,29].

All thermograms present a single loss in weight in a narrow range of temperatures, and this should be attributed to a relative uniformity in the chemical composition of the materials, indicating a rather pure and simpler material than that belonging to the first category; they are probably molecules of long-chain hydrocarbons.

### 3.2. FTIR Spectroscopy Analysis of Resins

Infrared spectroscopy was used to classify the different resins based on their spectra. Diterpenic, sequiterpenic and triterpenic resins were identified on the basis of different functional groups.

**Figure 2:**
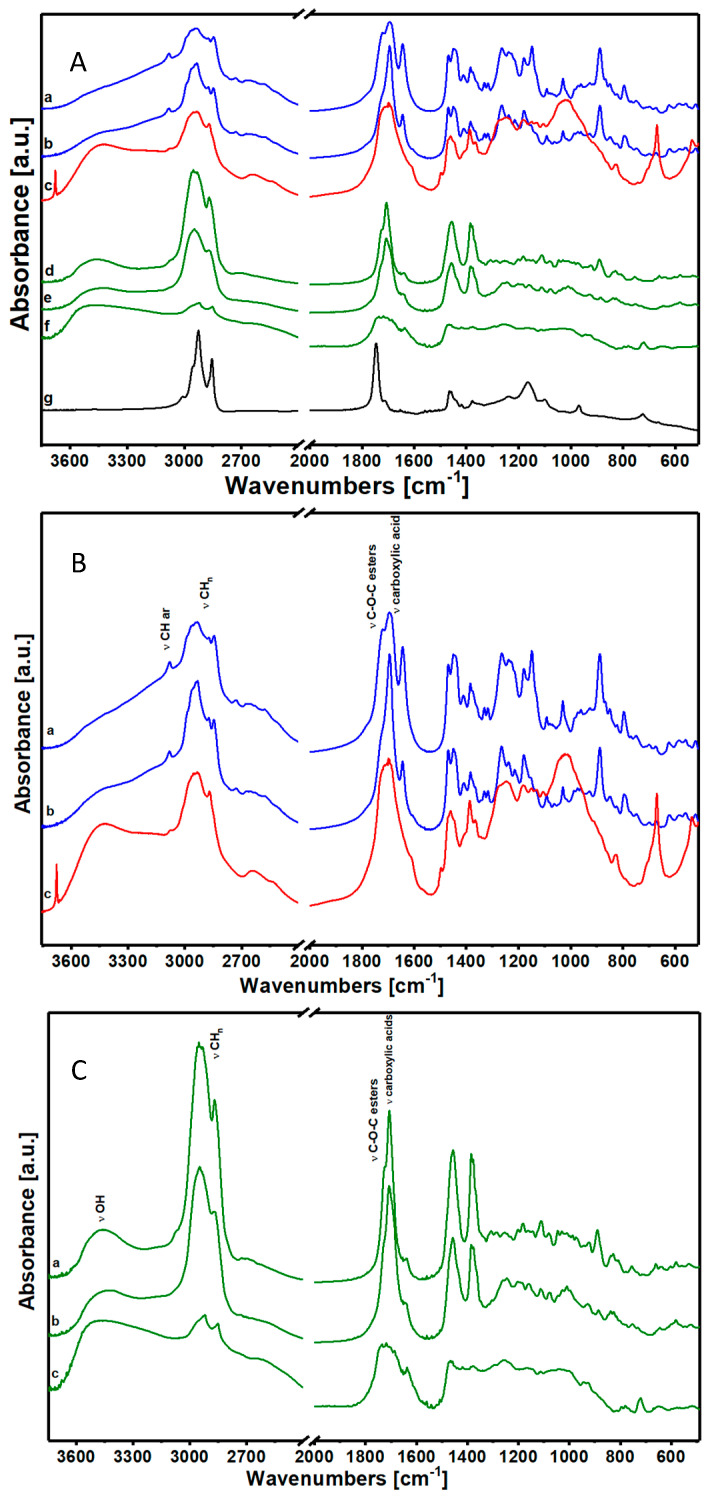
(**A**) FTIR spectra in air of the samples (a) Manila Copal, (b) Sandarac, (c) Colophony, (d) Dammar, (e) Mastic, (f) Shellac and (g) Flaxseed Stand Oil. (**B**) FTIR spectra in air of the samples (a) Manila Copal 60150, (b) Sandarac 60100 and (c) Colophony 60310. (**C**) FTIR spectra in air of the samples (a) Dammar 60000, (b) Mastic 60050 and (c) Shellac 60410.

According to the literature and considering the FTIR spectra, natural resins were classified as the (i) diterpenic class and the (ii) sesquiterpenic and triterpenic class [30,31,32]. Manila Copal, Sandarac and Colophony were classified in the first class, and their spectra are reported in Figure 2. Dammar, Mastic and Shellac were classified in the second class, as sesquiterpenic and triterpenic resins. Sesquiterpenic and triterpenic resins’ spectra are reported in Figure 2. The spectrum of flaxseed oil is also reported in Figure 2: this type of oil is used in Cultural Heritage combined with resins.

Even the FTIR spectra, in agreement with the literature, confirm the subdivision into two classes [30,31,32]. The first diterpenic class (Figure 2A; 2a Manila Copal, 2b Sandarac and 2c Colophony) and the second sesquiterpene and triterpene class (Figure 2A; 2d Dammar, 2e Mastic and 2f Shellac), together with the (Figure 2A; g) oil of seeds of flax, are represented in Figure 2A.

The diterpenic class, represented in Figure 2B, shows spectra that are groups linked to the presence of carboxylic acids and esters tied in aliphatic chains. The clear presence of the aryl group in the range of 3000–3100 cm^−1^ leads us to think that this is a very complex structure with aromatic rings isolated or condensed with aliphatic cyclic structures. Moreover, the presence of a broad band at high wavenumbers indicates formations of hydrogen bonds probably due to the interaction among carboxylic acids and to the consequent formation of bridge structures among the carboxyl groups. In addition to that, it is not excluded that these resins present, inside their structure, isolated atoms of sulfur or fluorine, probably linked to aromatic structures (in fact, the spectra show peaks related to mono- or di-substituted benzenes in the area of low wavenumbers). Unfortunately, due to the large overlap of signals in the fingerprint areas, it was not possible to make a precise assignment relative to the presence of these bonds.

The list of attributions of IR bands is shown in Table 1.

In Figure 2B, it can be seen that all the three materials exhibit the same spectral features: the vibrations of the CH bonds of the aliphatic groups (2800–3000 cm^−1^), the vibrations of the carbonyl of esters (1720 cm^−1^) and carboxylic acid (1695 cm^−1^), and that of the relative hydrogen bridges (broad band centered at 3000 cm^−1^), as well as the presence of the aromatic group vibrations (at 3080 cm^−1^). In the case of Colophony 60310 (Figure 2B(c)), these characteristic vibrations are a bit more hidden by the strong signals produced by the presence of an inorganic component.

The triterpene and sesquiterpene class is shown in Figure 2C: even the three materials—(a) Dammar, (b) Mastic and (c) Shellac—have vibrations of bonds related to the presence of ester (1730 cm^−1^) and carboxyl (1710 cm^−1^) groups, and of the vibrations of the CH bond of aliphatic chains [33].

However, they differ from the first class because of the absence of the aromatic group and the presence of vibrations of -OH bonds of alcoholic groups (at 3450 cm^−1^) [6].

As highlighted by the thermogravimetric analysis, in these samples we face a very different situation than the first sub-category where we were in the presence of aromatic molecules condensed together in structures that develop concentrically. On the contrary, this category has probably fewer complex structures, linearized on long aliphatic chains.

### 3.3. TGA-FTIR Analysis of Resin Degradation

The spectra recorded during the controlled degradation of the samples confirmed further division into the two classes of resins.

**Figure 3:**
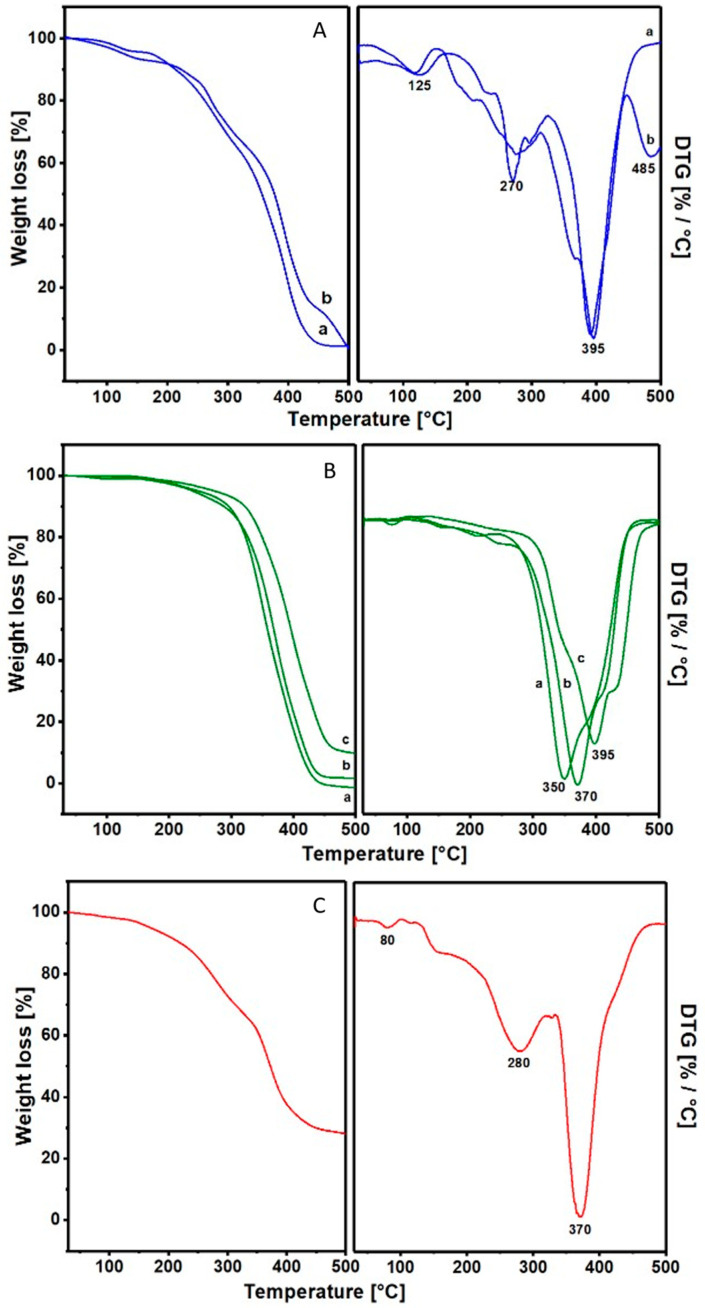
(**A**) TGA spectra in air of the samples (a) Sandarac 60100 and (b) Manila Copal 60150 at 140, 290 and 400 °C in thermal degradation. (**B**) TGA spectra in air of the samples (a) Dammar 60000, (b) Mastic 60050 and (c) Shellac 60410 at 250 and 390 °C in thermal degradation. (**C**) Thermogravimetric analysis of Colophony 60310 and the related derivative (DTG). On the right panels are the relative DTG analyses.

From FTIR analysis, Sandarac and Manila Copal, representatives of the first diterpenic class, in addition to similar weight losses in a similar temperature range, also have the same degradation behavior. This is shown in Figure 3A, where the spectra of (a) Sandarac and (b) Manila Copal are grouped at the degradation temperature relative to major losses in weight.

At 140 °C, we have a 5% loss in both resins in correspondence with a formation of free -OH, relative to the dehydration process.

The second temperature range illustrates the curve at 290 °C showing the lowering of the bands of the carbonyl groups.

Finally, at 400 °C, the curves show the slow disappearance of the double bonds and the start of the complete degradation of the structure of the resins.

The thermal degradation of the resins of the triterpene and sesquiterpene class is represented in Figure 3B.

Even Dammar (a), Mastic (b) and Shellac (c) have very similar degradation behaviors.

The spectrum at 250 °C represents the situation of the absorption just before the maximum fall of weight, where there is a formation of free -OH, with a consequent lowering of the band relative to the -OH of the alcohol groups.

At 390 °C, the spectrum of the Dammar resin (a) indicates that the structure is already almost completely degraded; in fact, the last maximum weight loss drops to 350 °C. The Mastic spectra (b) show the maximum weight loss at 370 °C, where the infrared spectra for these temperatures testify to the beginning of the structure collapse. Finally, the Shellac spectra (c) reveal that a serious decline has begun with a maximum of DTG to 390 °C.

The temperature ranges with the relative weight losses of all six resin samples, divided into two classes, are schematized in Table 2.

### 3.4. Thermogravimetric Analysis of Colophony 60310

The thermogravimetric analysis of Colophony 60310 highlights the presence of 30% of residual material at 500 °C; the result is shown in Figure 3C.

### 3.5. SEM/EDX Analysis of Colophony 60310

**Figure 4:**
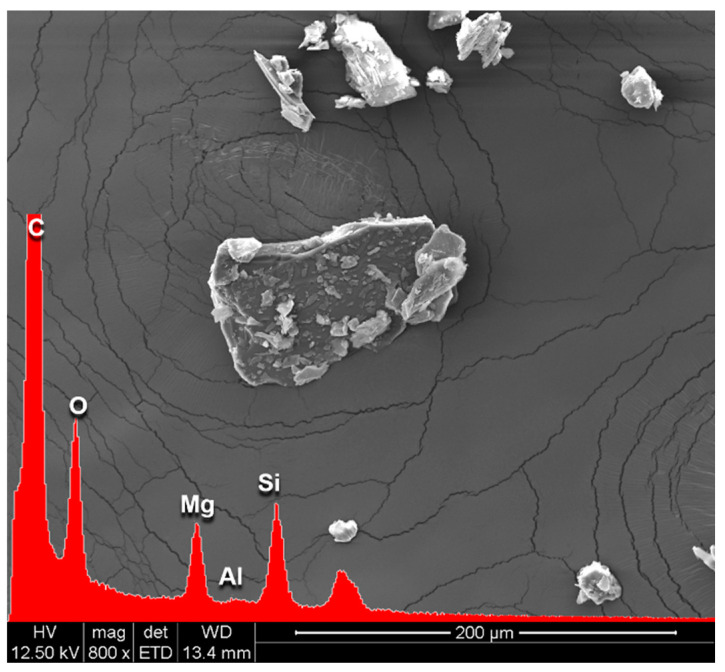
SEM image to 800× of Colophony 60310 and its EDX spectrum.

The elementary EDX analysis of Colophony 60310, shown in Figure 4, and the image acquired by a scanning electron microscope (SEM), detected the presence of 1.24% of Mg atoms, 0.03% of Al atoms and 1.62% of Si atoms (represented in Table 3). The elemental composition and particle shapes and dimensions are compatible with talc particles [34]. Talc was added as an additive in Colophony.

### 3.6. Spectroscopy Analysis of Colophony 60310

**Figure 5:**
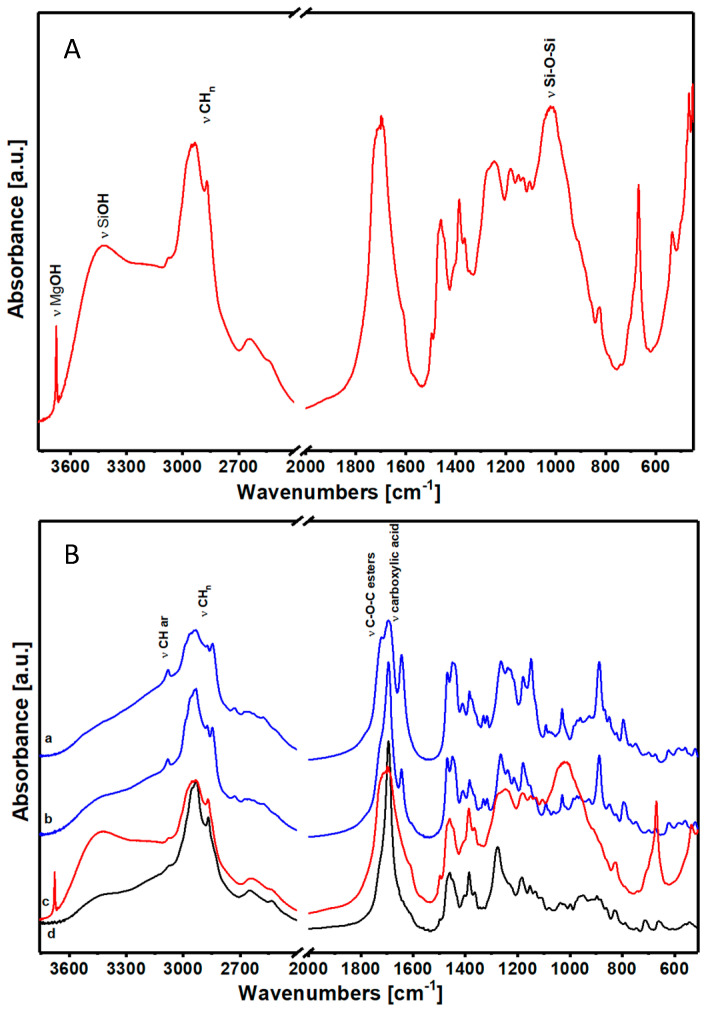
(**A**) FTIR spectra in air of the sample Colophony 60310. (**B**) FTIR spectra in air of the samples (a) Manila Copal 60150, (b) Sandarac 60100 and (c) Colophony 60310 with (d) Colophony 60300.

The spectra of Colophony 60310 are shown in Figure 5A. The presence of the inorganic component in the Colophony resin 60310 is demonstrated by the intense peak at 3676 cm^−1^, characteristic of stretching of the -OH free bond typically ascribed to the MgOH group and the stretching of the -OH bond interacting with the hydrogen bond of the SiOH group at 3430 cm^−1^. In the low wavenumbers area, there is an intense absorption at 1017 cm^−1^, which indicates the presence of O-Si-O bonds within the material. Finally, between 535 and 453 cm^−1^, there are three characteristic peaks of the inorganic component of the material. This inorganic additive is the lamellar phase of talc, which may have been added in the manufacturing processes as an anti-adhesive resin.

Comparing (c) Colophony 60310 with another one free from additives, Colophony 60300 (d), it is shown that Colophony indeed belongs to the first class of materials and, therefore, it is a diterpenic resin. This can be assumed by the similarity of functional groups and of their spectra to the (a) Manila Copal and (b) Sandarac resins, as shown in Figure 5B.

### 3.7. FTIR Analysis of the Degradation of Colophony 60310 and 60300

**Figure 6:**
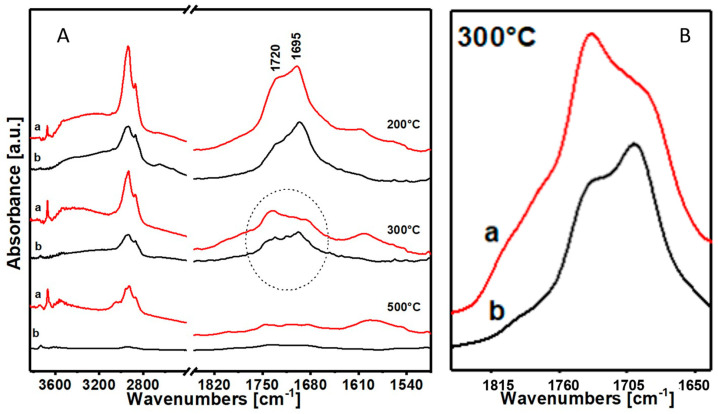
(**A**) FTIR spectra in air of the samples (a) Colophony 60310 and (b) Colophony 60300 at 200, 300 and 500 °C in thermal degradation; (**B**) inset of the region between 1840 and 1640 cm^−1^ at 300 °C, (a) Colophony 60310 and (b) Colophony 60300.

In Figure 6, FTIR spectra in air of the two resins, Colophony 60310 (a) and 60300 (b), subject to thermal degradation are reported and compared.

It can be verified that Colophony 60300 (b) presents a much more uniform and gradual degradative behavior: in fact, the peak intensities tend to uniformly decrease without variations in proportion. In contrast, Colophony 60310 (a), containing talc, undergoes an abrupt degradation of the structure with variation in the initial aspect ratio of the peak intensities related to the acid groups.

Comparing the spectra at 300 °C of the two resins, it is possible to see that in Colophony 60310, which contains the additive, the component which corresponds to the stretching of the carboxyl group has practically disappeared below the component of the ester group; in the spectrum at 300 °C of Colophony 60300, without an additive, the band relative to the carboxyl group stretching is still clearly visible. This highlights a catalytic effect on the degradation of the inorganic additive on the material that contains it.

### 3.8. FTIR Spectroscopy Analysis of Paint Layers of Resins/ZnO

**Figure 7:**
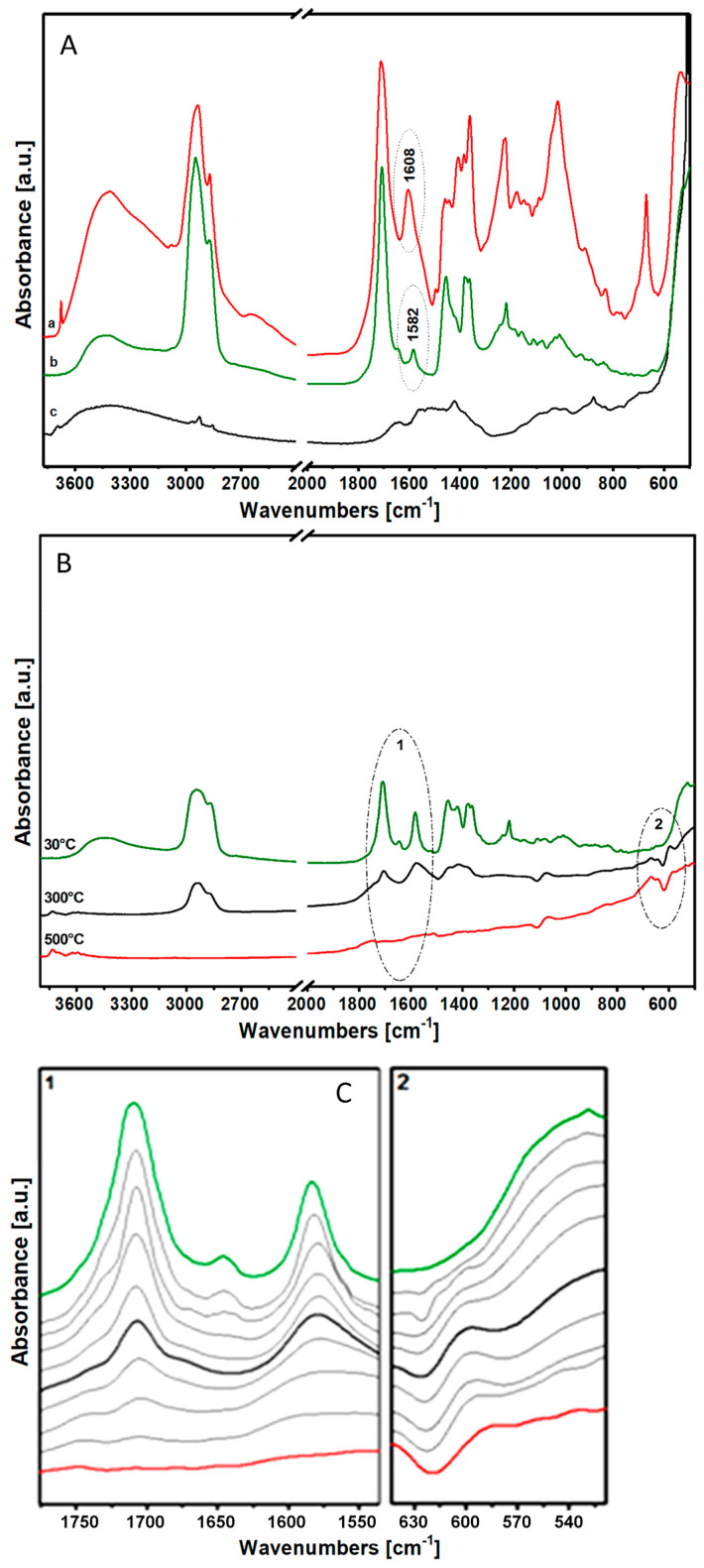
(**A**) FTIR spectra in air of the paint layer of (a) Colophony 60310/ZnO and (b) Mastic/ZnO (c indicates the spectrum of ZnO); (**B**) FTIR spectra in air of the paint layer of Mastic/ZnO at 30 (green line), 300 (black line) and 500 (red line) °C; (**C**) FTIR spectra in air of the paint layer of Mastic/ZnO, detailing the (1) carbon area and (2) oxalate formation area in thermal degradation. Grey lines represent intermediate temperature condition between 30, 300 and 500 °C.

Both layers of (b) Mastic/ZnO and (a) Colophony 60310/ZnO, as shown in Figure 7A, present characteristic absorptions relative to the metal oxides generated by the presence of the pigment, even at low wavenumbers (around 500 cm^−1^), in addition to the characteristic peaks of the component relative to the ligands. The most important feature is the intense absorption at around 1600 cm^−1^, especially considering the paint layer of (a) Colophony 60310/ZnO, relative to the formation of metal soaps, which is the cause of the degradation of the paint layers.

### 3.9. FTIR Analysis of the Thermal Degradation of the Paint Layer of Mastic/ZnO

During the thermal degradation of the paint layer of Mastic/ZnO, the spectra showed, in the range of the carbonyl (Figure 7B, circle 1), a uniform reduction of ester mixtures, aliphatic acids and alicyclic terpenes, with progressive disappearance of the conjugated double bonds C=C to 1640 cm^−1^ [11]. Even the band centered at 1580 cm^−1^, relative to the metallic soaps of zinc, progressively decreases in intensity with increasing temperature (Figure 7B, circle 1). It was finally shown that, with increasing temperature, there is a gradual formation of a new component to about 600 cm^−1^, highlighted in zone 2 in Figure 7B, relative to the formation of metal oxalates, responsible for the impairment of the paint film.

### 3.10. FTIR Spectroscopy Analysis of the Photo-Oxidative Degradation of the Paint Layer of Mastic/ZnO

**Figure 8:**
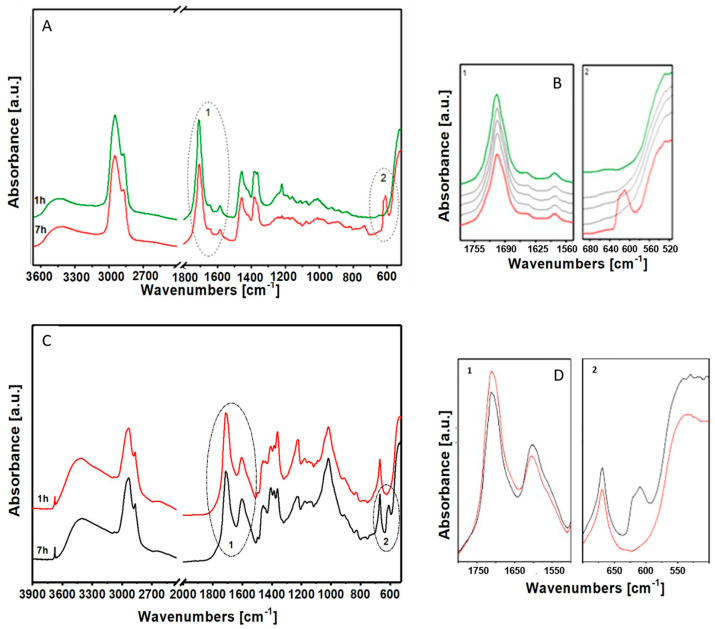
(**A**) FTIR spectra in air of the paint layer of Mastic/ZnO after 1 h and 7 h of photo-oxidative degradation; (**B**) details of the (1) carbon area and (2) oxalate formation area, the grey curves show the course of the materials in the time between 1 h and 7 h, the spectra are 1.5 h apart. (**C**) FTIR spectra in air of the paint layer of Mastic/ZnO after 1 h and 7 h of photo-oxidative degradation; (**D**) details of the (1) carbon area and (2) oxalate formation area.

The spectra in air of the paint layer of Mastic/ZnO after one hour and after seven hours of exposure to a UV lamp are shown in Figure 8A.

Observing the stretching of the carbonyl zone (Figure 8A, circle 1), a decrease in the bands related to ester and aliphatic acid mixtures and terpenic alicyclic acids can be seen, as in the case of heat treatment.

On the contrary, the band at 1640 cm^−1^, relative to the conjugated double C=C bonds, is still slightly visible. Instead, the band relative to the metal soaps almost imperceptibly diminishes. Finally, in the second range (Figure 8A, circle 2), the formation of a new band centered at 600cm^−1^ is visible after seven hours, relative to the formation of zinc oxalates.

### 3.11. FTIR Spectroscopy Analysis of the Photo-Oxidative Degradation of the Paint Layer of Colophony 60310/ZnO

The composition and degradation behavior of the paint layer of Colophony 60310/ZnO under the applied aging conditions is described and discussed, in the following, starting from the concordant results reported in Figure 8B. The spectra of the paint layer after 7 h of solar-simulated aging present a decrease in the intensity of the bands related to ester mixtures, aliphatic acids and alicyclic terpenes in the area of carbonyl absorptions (Figure 8B, circle 1), accompanied by a growth of the band related to the formation of metal soaps. The last area (Figure 8B, circle 2) reports, after seven hours of exposure to UV light, the formation of a signal around 600 cm^−1^, identifying zinc oxalate.

The preparation of Colophony 60310 and zinc oxide presents, therefore, unlike the research of Mastics, an increase in the formation of metal soaps after seven hours of photo-oxidative degradation.

## 4. Discussion

The interactions of the acid groups of the binder with the metal ions of the pigment through hydrolysis reactions leading to the formation of metal soaps are, in many cases, the main factors responsible for the paint film deterioration. TGA and FTIR analyses have been applied in order to monitor the presence of acid groups—and, in general, the chemical nature of the studied materials. So, it was possible to classify the resins into two categories that showed unexpected homogeneity in degradation mechanisms and in the presence of the same functional groups.

Specifically, by coupling FTIR techniques to thermogravimetric analysis, it was possible not only to qualitatively determine the acid groups present inside the binders but also to observe the behavior during simulated thermal degradation treatments from room temperature up to 500 °C.

Particular attention was paid to Colophony 60310 resin, which showed a large fraction of residual material (about 30% by weight) at 500 °C in TGA analysis; to confirm this, FTIR analysis of the spectra showed the presence of inorganic material additives, which has finally been identified as talc.

This inorganic fraction systematically alters binder reactivity toward the formation of metal soaps, increasing the propensity to thermal degradation. To confirm the particular reactive capacity, Colophony 60310 was confronted with another Colophony not containing inorganic extra phases. It was then established that the inorganic component within the resin matrix leads to the modification of its chemical and physical characteristics, altering the degradative behavior.

Based on the experimental results, it was possible to choose the binders to be mixed with zinc oxide in paint layers. We verified by FTIR spectroscopy the mode of degradation of the layer and the possible formation of new components through heat treatment and photo-oxidation simulating real conditions of degradation.

The paint layers containing Mastic and Colophony 60310, presented before being subjected to degrading treatment in the presence of metal soaps, highlighted a high interaction among the phases of the film, probably due to the high reactivity of ZnO.

In particular, the preparation of Colophony 60310 and zinc oxide highlighted the formation of zinc oxalate, which is one of the main causes of degradation of the paint layers, in both degradative treatments. In particular, the Colophony resin with an additive, unlike the Mastic triterpene resin, exhibited additional metal soap development after 7 h of photo-oxidative treatment.

**Figure 9:**
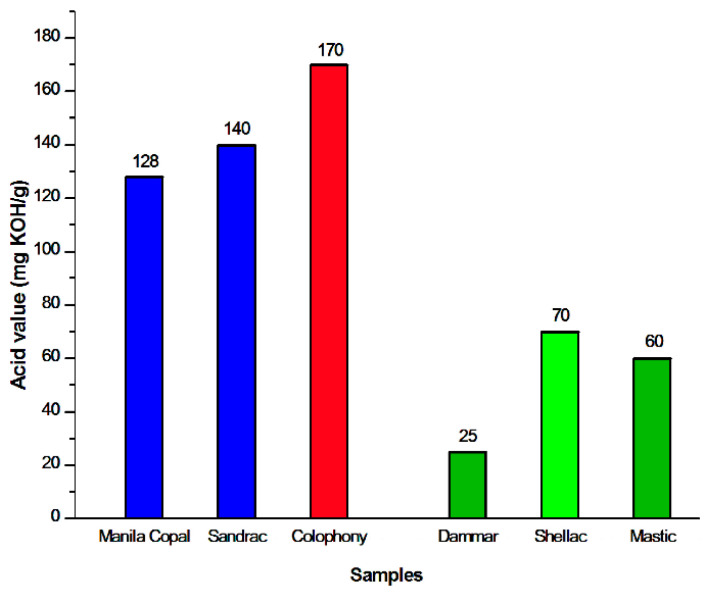
Histogram of acid value (mg KOH/g) of resins.

The mechanisms that occur within the interface binder/pigment are not yet completely understood, but in this work, it has been shown that the degree of binder acidity (Figure 9), which is given both by the intrinsic properties of the binder material and by the presence of an additive, varies the type of interaction of the resin with the cationic components of the metallic pigment in a substantial way. Moreover, this leads to the formation of new species, such as oxalates and metal soaps, which cause the surfaces of works of art to disintegrate.

## Figures and Tables

**Table 1:** List of attributions of infrared bands.

Wavenumbers (cm^−1^)	Assignment
453–535	peaks of talc
600	Zn oxalates
1017	ν O-Si-O group
1580–1600	Zn soaps
1640	ν C=C
1695–1710	ν carboxylic acid
1720–1730	ν carbonyl of ester
2800–3000	ν alkyl group
3100	ν aryl group
3430	ν-OH interacting with hydrogen bond of SiOH group
3450	ν alcoholic groups
3676	ν-OH in MgOH

**Table 2:** Range of temperature and weight losses of resin samples.

	Temperature (°C)				
	50–200	200–300	300–500		
Manila Copal	10%	20%	60%	Loss in Weight (%)	95%
Sandarac					
Colophony			40%		70%
Shellac	5%		90%		95%
Dammar					
Mastic					

**Table 3:** Atomic percentages of C, O, Mg, Si and Al contained in the sample Colophony 60310.

	Elements (%)				
Sample	C	O	Mg	Si	Al
Colophony 60310	83.80	13.30	1.20	1.60	0.03

## Data Availability

The original contributions presented in the study are included in the article, further inquiries can be directed to the corresponding author.
